# Spin-controlled topological phase transition in non-Euclidean space

**DOI:** 10.1007/s12200-024-00110-w

**Published:** 2024-03-19

**Authors:** Zhuochen Du, Jinze Gao, Qiuchen Yan, Cuicui Lu, Xiaoyong Hu, Qihuang Gong

**Affiliations:** 1 State Key Laboratory for Mesoscopic Physics and Department of Physics, Collaborative Innovation Center of Quantum Matter and Frontiers Science Center for Nano-Optoelectronics, Beijing Academy of Quantum Information Sciences, Peking University, Beijing, 100871 China; 2 Key Laboratory of Advanced Optoelectronic Quantum Architecture and Measurements of Ministry of Education, Beijing Key Laboratory of Nanophotonics and Ultrafine Optoelectronic Systems, School of Physics, Beijing Institute of Technology, Beijing, 100081 China; 3 Peking University Yangtze Delta Institute of Optoelectronics, Nantong, 226010 China; 4 Collaborative Innovation Center of Extreme Optics, Shanxi University, Taiyuan, 030006 China; 5 Hefei National Laboratory, Hefei, 230088 China

**Keywords:** Topological phase transition, Non-Euclidean space, Möbius ring, Spin-locked effect

## Abstract

Modulation of topological phase transition has been pursued by researchers in both condensed matter and optics research fields, and has been realized in Euclidean systems, such as topological photonic crystals, topological metamaterials, and coupled resonator arrays. However, the spin-controlled topological phase transition in non-Euclidean space has not yet been explored. Here, we propose a non-Euclidean configuration based on Möbius rings, and we demonstrate the spin-controlled transition between the topological edge state and the bulk state. The Möbius ring, which is designed to have an 8π period, has a square cross section at the twist beginning and the length/width evolves adiabatically along the loop, accompanied by conversion from transverse electric to transverse magnetic modes resulting from the spin-locked effect. The 8π period Möbius rings are used to construct Su–Schrieffer–Heeger configuration, and the configuration can support the topological edge states excited by circularly polarized light, and meanwhile a transition from the topological edge state to the bulk state can be realized by controlling circular polarization. In addition, the spin-controlled topological phase transition in non-Euclidean space is feasible for both Hermitian and non-Hermitian cases in 2D systems. This work provides a new degree of polarization to control topological photonic states based on the spin of Möbius rings and opens a way to tune the topological phase in non-Euclidean space.

**Graphical abstract:**

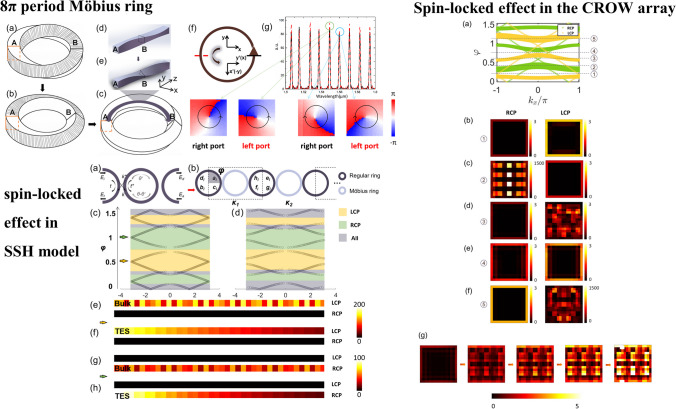

**Supplementary Information:**

The online version contains supplementary material available at 10.1007/s12200-024-00110-w.

## Introduction

In the last decade, topological photonics has provided a robust platform to study light-matter interactions and optical devices [1–5]. Modulation of topological phase transition is a focus of research in the field of topological photonics, and has been realized in Euclidean systems, such as topological photonic crystals, topological metamaterials, and coupled resonators [6, 7]. However, the topological configurations in optics are always based on Euclidean components, which does not make full use of the curvature in space, and there remains no counterpart in non-Euclidean configurations of topological photonics. Euclidean geometry applies to a plane and when the curvature of a surface is not 0, it must be described by non-Euclidean geometry. The surface of the Mobius ring can be seen as a tape twisted π and formed end to end, which is a typical non-Euclidean geometry model. Non-Euclidean photonics has received intense attention recently [8–17], and this has greatly enriched the research of the topological world. However, topological phase transition in non-Euclidean space has not yet been explored.

**Fig. 1:**
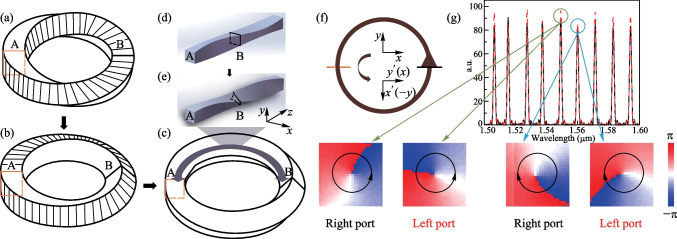
**a** A regular Möbius ring with 4π period. **b** 8π period Möbius ring. **c** 8π period Möbius ring with length/width adiabatic evolution. **d** Length/width adiabatic evolution in straight waveguide. **e** Length/width adiabatic evolution in straight waveguide with twist operation. **f** Light travel through one turn in the 8π period Möbius ring. **g** Transmittance spectra for 8π period Möbius ring of right port (black line) and left port (dotted red line), as well as the phase distribution along the propagation direction. Transmittance spectra means the ratio of the electric field intensity that can be transmitted through the port to the incident electric field intensity, and its changing with the wavelength

In this work, we propose a novel Möbius ring for the study of topological phase transition, where the spin is adopted as a new degree of freedom to control topological edge states. The Möbius ring is designed to have an 8π period ring (8PMR) and a π/2-twist, supporting the spin-locked effect, as shown in Fig. 1. This Möbius ring can be recognized as a waveguide, which has a square cross section in the Möbius twist beginning and the length/width evolves adiabatically along the loop, converting transverse electric (TE) and transverse magnetic (TM) waveguide modes. The 8PMRs are adopted to construct both a one-dimensional (1D) Su–Schrieffer–Heeger (SSH) model and two-dimensional (2D) coupled resonant optical waveguide (CROW) arrays. These configurations based on the spin-locked 8PMR can only support the topological edge states (TES) excited by left(right)-circularly polarized light, while the topological modes excited by the right (left)-circularly polarized light are forbidden. The phase transition from the topological edge state to the bulk state can be conveniently realized by controlling circular polarizations for both Hermitian and non-Hermitian cases. This work provides a novel degree of freedom for tuning topological edge states based on Möbius rings, and paves the way for studying topological phase in non-Euclidean space.

## Design of 8π period Möbius ring

The Möbius resonant ring is usually considered as a non-Euclidean ring having a twist period of 4π, i.e., an object will return to its starting position after two-turns moving on the ring surface, as shown in Fig. 1a. If we define a starting cross section of the Möbius ring, there will be a π-twist operation of the section during the process of the start part and end part connections. This is a “regular” Möbius, which has attracted lots of attention because of its unique mathematical and physical properties. However, there is always one missing point: the twist’s angle is not necessarily an integer multiple of π, but also can be a half integer multiple of π.

To satisfy the resonance condition of circular polarized light, the values of electric field components in the *x* and *y* directions should convert to each other. The Berry connection, which represents the change between the two eigenstates by a matrix in the adiabatic process, can reveal the conversion. In the process of adiabatic evolution, the Berry connection is defined as

1 $$\begin{array}{c}\gamma \left({\theta }_{0}\right)=\text{i}\int \langle \psi \left({\theta }_{0}\right)|\frac{\partial }{\partial {\theta }_{0}}|\psi \left({\theta }_{0}\right)\rangle {\text{d}}{\theta }_{0},\end{array}$$

where ${\theta }_{0}$ is a parameter of the Hamiltonian $H$ of the system, representing the accumulated dynamic phase for traveling one turn. As there are two eigenstates, $\gamma \left({\theta }_{0}\right)$ is a two-dimensional matrix, which is $$2\mathrm{\pi i}\left[\begin{array}{cc}1& -1\\ 1& 1\end{array}\right]$$. In this case, the TE mode and TM mode can be converted to each other, producing an interesting phenomenon. Here, the TE mode indicates that the electric field direction is parallel to the *x*-axis, and the TM mode indicates that the electric field direction is parallel to the *y*-axis. If a vector $[a; b]$ is used to represent the initial mode, after the light travels for one cycle, the mode in the Möbius ring becomes $[b* {{\text{e}}}^{{\text{i}}{\theta }_{0}}; -a*{{\text{e}}}^{{\text{i}}{\theta }_{0}}]$. Moreover, due to the mutual transformation of TE and TM modes, the equations can be read as follows:

2 $$\begin{aligned}&b=-a*{{\text{e}}}^{{\text{i}}{\theta }_{0}},\\& a= b* {{\text{e}}}^{{\text{i}}{\theta }_{0}}= -a*{{\text{e}}}^{{\text{i}}2{\theta }_{0}}.\end{aligned}$$

Then the parameter ${\theta }_{0}$ should meet the following condition,

3 $$\begin{array}{c}{\theta }_{0}=\left({\text{n}}+\frac{1}{2}\right)\uppi , {\text{n}}\in Z+.\end{array}$$

Here, we can simplify the mode of $[a; b]$ as $[1; -{\text{i}}*{(-1)}^{n}]$, which is the resonance condition. If *n* is an even number, the mode becomes $[1, {\text{i}}]$, which indicates that the Möbius ring only supports left-spin (left circular polarized) light (LCP). While if *n* is an odd number, the mode becomes $[1, -{\text{i}}]$, which indicates that the Möbius ring only supports right-spin light (RCP).

Therefore, in this letter, a Möbius resonant ring with a π/2-twist operation is designed, and its spin-locked condition is verified. Since this kind of Möbius resonant ring can be considered as a waveguide, like a regular resonant waveguide ring [18], we take a Möbius ring made of silicon material as an example to specifically describe the behaviors. For a regular single-mode silicon waveguide, whose length is generally 500 nm and the width is 220 nm, its beginning cross section and end cross section cannot match or exactly connect after a π/2-twist operation. Therefore, in order to implement the matching in a π/2-twist Möbius ring, we make both the length and width sizes of the Möbius ring 500 nm; this can still support fundamental TE/TM mode. The schematic is shown in Fig. 1b. When the light travels in this 8PMR for one cycle, see Fig. 1f, both the *x*-axis and *y*-axis turn clockwise 90°, causing the *x* direction to become the *y* direction, and the *y* direction to become the *x* direction. That is, the original TE mode changes into TM mode and vice versa.

However, even though the spin-locked effect works in theory, the simulation results show that such a Möbius ring is still unable to achieve spin lock. When the sizes of length and width in 8PMR are equal, the TE mode and TM mode degenerate, and the two modes cannot be distinguished during the ring twist process. To break the degeneracy, we design so that the length and width of 8PMR change adiabatically during the twist process. The details can be found in Supplementary Materials I (SM I). A straight waveguide shown in Fig. 1d is used to clearly demonstrate the evolution process. When the cross-section A evolves to cross-section B, the length of the waveguide changes from 500 to 300 nm, then back to 500 nm; and the width changes from 500 to 700 nm, then back to 500 nm. To compensate for the difference of dynamic phases in the evolution of length and width, the length is changed from 500 to 700 nm and back to 500 nm when it evolves backward from cross-section B. The width changes correspondingly, i.e., from 500 to 300 nm and back to 500 nm. After this adiabatic evolution and π/2-twist operation (Fig. 1e), a suitable straight waveguide has been achieved. If the ending and starting cross sections of the straight waveguide connect, the TE and TM modes can break the degeneration, and the final 8PMR is formed as shown in Fig. 1c.

We further verify the spin-locked property. The radius of the Möbius ring is set to be 4 μm, which meets the adiabatic evolution conditions, and with π/2-twist operation. The injected light source has a basic TE mode at the starting cross section of 8PMR. Four monitors are placed at the symmetric positions of the ring, top, bottom, left, and right respectively. However, the Möbius ring data obtained from these monitors make sense only on the starting and middle sections, where the length and the width are equal. The two monitors’ data on transmittance are shown in Fig. 1g. The solid black line represents the starting-position transmission spectrum, the dotted red line represents the middle-position transmission spectrum. If we determine a wavelength from the ring’s free spectral range (FSR), for example 1549 nm circled in green, the phase of the waveguide mode has anti-clockwise distribution, no matter whether in the left or right monitor. Similarly, if we select 1559 nm, circled in blue, the phase of the waveguide mode always has clockwise distribution in these two monitors. Therefore, the Möbius ring can have the property of spin lock based on specific twist direction or excitation wavelength. In addition, it should be pointed out that for this kind of Möbius ring, involving adiabatic evolution, the radius of the ring is generally required to be large enough to ensure the process. In the case that the radius of the Möbius ring is small, for example, 3 μm or less, the 8PMR cannot realize the spin lock of specific circular polarized light, but the lock of a certain linear polarized light. Therefore, an additional dynamic phase $\delta$ should be introduced to indicate whether the adiabatic evolution is met. When $\delta$ is zero, it represents the ring with adiabatic evolution and spin lock. When $\delta$ is π, it represents the ring with non-adiabatic evolution and just linearly-polarized light lock. When $\delta$ has other values, the Möbius ring may have unexpected polarized direction locking. The detailed descriptions are in SM II. So far, we have successfully designed a spin-locked Möbius ring and verified its non-Euclidean characteristics.

## SSH topological configuration with 8PMR

**Fig. 2:**
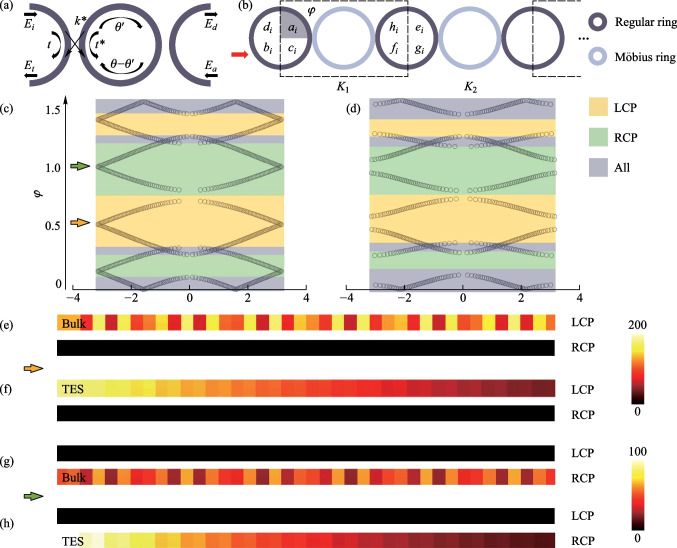
**a** Add-drop type micro rings units. **b** SSH model composed of add-drop type units. The propagation direction in the main rings is specified as clockwise. **c** Band structure at *k*_1_ = *k*_2_. **d** Band structure at *k*_1_ < *k*_2_. **e**, **f** Bulk state and TES, excited by optical frequency in yellow regions in **c**, **d**. **g**, **h** Bulk state and TES, excited by optical frequency in green regions in **c** and **d**

To explore the influences of non-Euclidean structure on the topological photonics, the kind of Möbius ring described above is introduced into the optical topological configurations. Assuming that the 8PMR can have either left-spin lock or right-spin lock in a certain frequency, we can take the π/2-twist 8PMR as an example to illustrate behavior in non-Euclidean topological photonics. The SSH model, as one of the most typical configurations in topological photonics [19–21], is taken to illustrate the non-Euclidean characteristics of 8PMR. The Hamiltonian of the SSH model is read as Eq. (4).

4 $$\begin{array}{c}\widehat{H}={k}_{1}\sum _{m=1}^{N}\left(\left|m,B> <m,A\right|+h.c.\right)+{k}_{2}\sum _{m=1}^{N-1}\left(\left|m+1,A> <m,B\right|+h.c.\right),\end{array}$$

where the intra-coupling strength is represented by *k*_1_ and the inter-coupling strength is represented by *k*_2_. *m* represents each site number of units and *N* represent the total site number of units. The method of bulk-edge correspondence (BEC) is used to describe the topological phase and its phase transition. When the band gap is topological, there are topological edge states in the band gap of the band structure, which mean that some eigenwave functions of the system are concentrated near the boundary and quickly decay away from the boundary. And when the band gap is trivial, there are only bulk states in the band structure, which means that some eigenwave functions of the system are concentrated near the boundary and quickly decay away from the boundary. When *k*_1_ < *k*_2_, the optical system is topological and generates TES. Consideration of 8PMR as a link ring in add-drop type micro rings (the unit in SSH) is shown in Fig. 2a. The detailed derivation on the coupling relationship is provided in SM III, including handling of π/2-twist 8PMR as a matrix of $$\frac{\sqrt{2}}{2}\left[\begin{array}{cc}1& -1\\ 1& 1\end{array}\right]$$.

Based on the units of add-drop type micro rings, we arrange them according to the SSH model (Fig. 2b), calculate the energy band structures and corresponding field distributions. To simplify, *φ* is defined as the phase accumulation by the light field through 1/4 of the main ring, to represent different optical frequencies. For a fixed-size resonant ring, once the reference frequency is determined, the value of *φ* can be considered as zero. As the frequency increases, the *φ* value increases accordingly. The transfer matrix method (TMM) is used to obtain the bulk energy band structure [22], the results are shown Fig. 2c, d. The *x* coordinate represents the Floquet period boundary condition in the *x* direction, and the *y* coordinate represents the optical frequency measured by *φ*. Because the coupling coefficient $k$ and transmission coefficient $t$ have the relationship of ${k}^{2}+{t}^{2}=1$, and it is convenient to change $t$ in experiments (for example, it can be obtained by measuring the transmission of waveguide), we here change parameter $t$ to regulate the energy band structures. In the case of *k*_1_ = *k*_2_ (Fig. 2c, *t*_1_ = *t*_2_ = 0.5), the band gap is closed. In the case of *k*_1_ < *k*_2_ (*t*_1_ = 0.7, *t*_2_ = 0.3), the band gap reopens, shown in Fig. 2d. Moreover, the configurations can support different spin-direction light under incident light excitation with different frequency, and the results are the same as in the simulation. The yellow regions represent supporting LCP light, the green regions represent supporting RCP light, and the maroon regions represent supporting both LCP and RCP light. Further results on the field distributions of each configuration with finite units are as follows under different spin-direction and frequency excitation. The incident light excites the ${b}_{i}$-port on the boundary. The value of *φ*, corresponding to the excited frequency, in the yellow region is 0.581 and in the green region is 1.260. In the critical case, there is bulk state under LCP light excitation while the mode is forbidden under RCP light excitation, shown in Fig. 2e; and there is bulk state under RCP light excitation while the mode is forbidden under LCP light excitation, shown in Fig. 2g. In the topological case, there is a TES under LCP light excitation, shown in Fig. 2f, and under RCP light excitation, shown in Fig. 2h. While the RCP light, shown in Fig. 2f, and LCP light, shown in Fig. 2h, cannot excite the optical mode due to spin-locked limit, which is very different from the regular Euclidean rings. From the above analysis, we conclude that the TES generation can be affected by the spin direction of incident light after introducing the non-Euclidean structure of the Möbius ring. Even if the configuration is topological, the TES will not be displayed without a matched excitation incident light. This provides a new degree of freedom for regulating the TES in the non-Euclidean space.

## Coupled resonator optical waveguide arrays with 8PMR

**Fig. 3:**
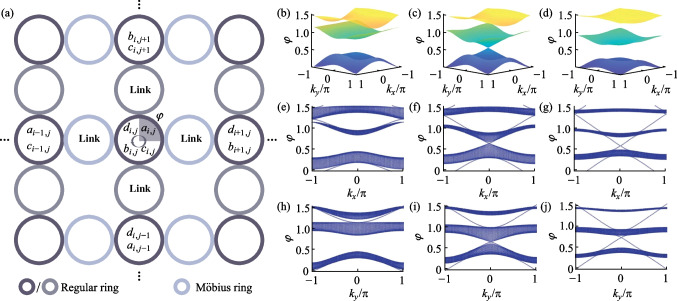
**a** CROW arrays with π/2-twist 8PMR and regular rings as the link rings; the main rings are all composed of the regular rings. The propagation direction in the main rings is specified as clockwise. **b**–**d** Bulk band structures arranged in the sequence *t* = 0.7, *t* = $\sqrt{2}-{1}$ and *t* = 0.2. **e**–**g** Projective band structures arranged in the sequence *t* = 0.7, *t* = $\sqrt{2}-{1}$ and *t* = 0.2 with Floquet periodic boundary condition in the *x* direction. **h**–**j** Projective band structures arranged in the sequence *t* = 0.7, *t* = $\sqrt{2}-{1}$ and *t* = 0.2 with Floquet periodic boundary condition in the *y* direction

Furthermore, the novel performance of the Möbius ring in the CROW array is calculated. Similarly, the π/2-twist 8PMR is considered as a link ring in the CROW array; the coupling relationship in a unit is explained in SM IV. In addition, in order to highlight the spin-locked effect of the Möbius ring and clearly distinguish the difference between field distributions under left-spin and right-spin light excitation, the 8PMR acts as the link ring only in the *x* direction. The regular resonant ring acts as the link ring in the *y* direction. The schematic is provided in Fig. 3a. The coupling relationship of add-drop micro ring configuration with the regular ring as the link ring is also supplied in SM V. The TMM is used to calculate the energy band structures with different coupling coefficients and is used to regulate the optical transmission in CROW arrays. We also change the parameter $t$ to regulate the band structures. In the case of *t* = 0.7, as shown in the Fig. 3b, there is a band gap in the bulk band. As the *t* value decreases to $\sqrt{2}-{1}$, the band gap is closed and the Dirac point appears, see Fig. 3c. This is a critical topological phase transition in CROW arrays. If we continue to decrease *t* value, the closed band gap reopens. Figure 3d demonstrates the bulk band structure in the case of *t* = 0.2. To observe the TES, projective bands are calculated by using an open boundary in one direction and a periodic boundary in the other. Projective bands with Floquet periodic boundary condition in the *x* direction and the *y* direction are obtained and demonstrated in Fig. 3e–g and h–j respectively, in the sequence *t* = 0.7, *t* = $\sqrt{2}-{1}$ and *t* = 0.2. In projective bands, there is an obvious Dirac point in the case of *t* = $\sqrt{2}-{1}$. After the topological phase transition, i.e., *t* = 0.2, the TES can be excited.

**Fig. 4:**
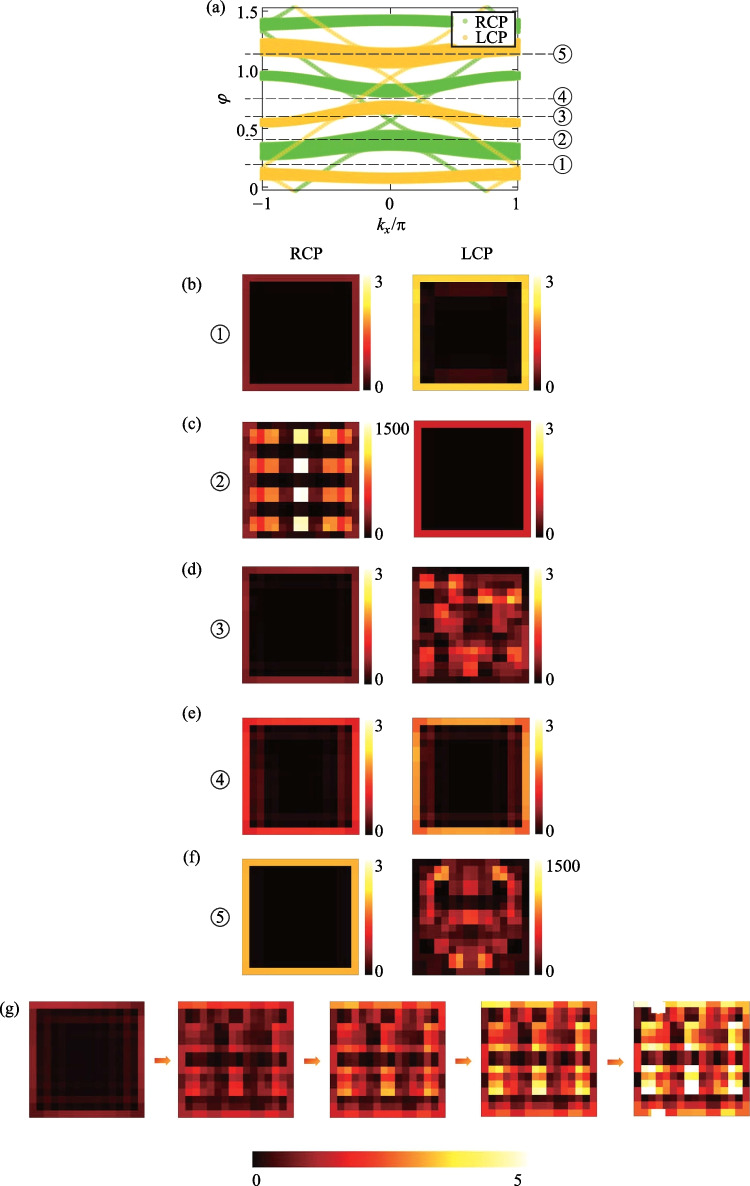
**a** Comparison of projective band structures of CROW arrays composed of π/2-twist 8PMR (green) and − π/2-twist 8PMR (yellow) for the Hermitian case. **b**–**f** Field distributions under either RCP or LCP light excitation (with each main ring abstracted as a color block); the *φ* values are selected in the sequence 0.2, 0.4, 0.63, 0.78, and 1.2, corresponding to the dotted lines in **a**. **g** Hybrid process in which the boundary state is gradually transformed into the bulk state with the proportions of left(right)-circularly polarized light in the input light being respectively 100% (0%), 70% (30%), 50% (50%), 30% (70%), and 0% (100%)

We further research the non-Euclidean characteristics of topological photonics, and the field distributions in different cases are obtained and shown in Fig. 4. Here, the projective band structures with π/2-twist 8PMR and − π/2-twist 8PMR are compared in Fig. 4a in the case of *t* = 0.2. The Floquet periodic boundary condition is in the X direction. The greens represent the bands with π/2-twist 8PMR, which result in locked RCP light under a certain frequency excitation. The yellow represents the bands with − π/2-twist 8PMR, which lock LCP light under the same frequency excitation. Five different *φ* values are selected to show the differences, in the sequence 0.2, 0.4, 0.63, 0.78, and 1.2. Figure 4b–f demonstrate the field distributions under either RCP light or LCP light excitation. In the case of *φ* = 0.2 and *φ* = 0.78, regardless of the spin of the light that excites the configuration, the TES can be generated. The two projective bands at this frequency are corresponding to a topological band, causing simultaneous TES generation. However, in the case of *φ* = 0.4, there is TES under LCP light excitation and a bulk state under RCP light excitation, due to the topological band in the LCP light case and the bulk band in the RCP light case under the same frequency of light excitation. Moreover, the calculated field intensity of the bulk state is much greater than TES, which illustrates the non-negligible bulk protection. Similarly, in the case of *φ* = 0.63 and *φ* = 1.2, the same reasoning can be used to analyze the TES under RCP light excitation and bulk state under LCP light excitation.

**Fig. 5:**
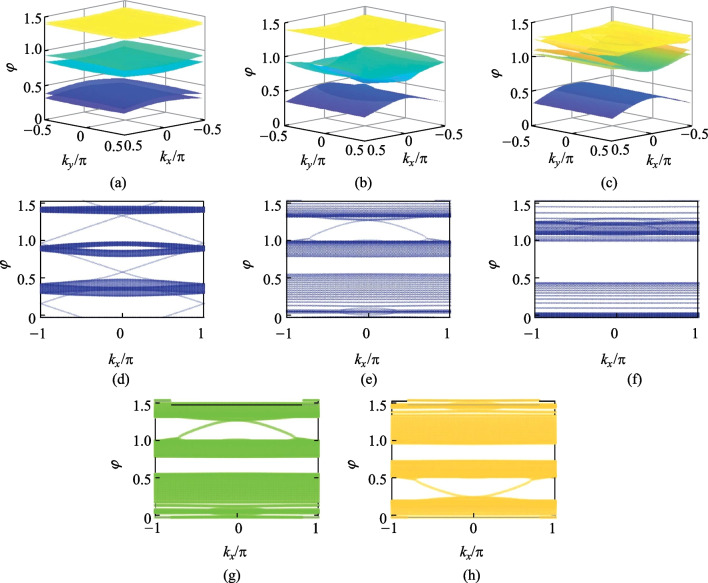
**a**–**c** Bulk band structures arranged in the sequence *g* = 0, *g* = 0.84, and *g* = 1.35 with Floquet periodic boundary condition in *x* direction, where *g* is the amount of the gain. **d**–**f** Projective band structures arranged in the sequence *g* = 0, *g* = 0.84, and *g* = 1.35 with Floquet periodic boundary condition in the *x* direction. **g**, **h** Comparison of projective band structures of CROW arrays composed of π/2-twist 8PMR (green) and − π/2-twist 8PMR (yellow) for the non-Hermitian case

Non-Hermitian conditions are introduced when the system is in a topological state with $t$ = 0.2. The same amount of gain $g$ and loss $-g$ are added to the main rings in turn. In the case of *g* = 0, as shown in Fig. 5a, the configuration is topological. As the *g* value increase to 0.84, the band gap is closed and the Dirac point appears, see Fig. 5b. If we continue to increase the *g* value, the closed band gap reopens. Figure 5c demonstrates the bulk band structure in the case of *g* = 1.35. To observe the TES, projective bands are calculated by using an open boundary in one direction and a periodic boundary in the other. Projective bands with Floquet periodic boundary condition in the *x* and *y* directions are shown in Fig. 5d–f, in the sequence *g* = 0, *g* = 0.84, and *g* = 1.35. In projective bands, there is an obvious Dirac point in the case of $\text{g }\text{= 0.84}$. Figure 5g, h demonstrate that the configuration can only support the topological edge states (TES) excited by left(right)-circularly polarized light, and meanwhile the topological modes excited by the right(left)-circularly polarized light are forbidden. The greens represent the band with π/2-twist 8PMR, which means that RCP light is locked and the yellow represent the band with − π/2-twist 8PMR, which means that LCP light is locked.

From the above discussions, it is clear that the Möbius ring can display the expected spin-locked effect in both 1D and 2D topological configurations in both Hermitian and non-Hermitian cases, which is accompanied by a multiplexing function due to excitation of TES based on spin-light.

## Conclusion and discussion

In conclusion, we have proposed an 8π period Möbius ring with spin-locked behavior, which means that polarized lights in the *x* and *y* directions can be interconverted. We have further verified the spin-locked effect in SSH model and CROW arrays to demonstrate the applications of 8PMR. These configurations based on the left (right)-spin-locked 8PMR can only support the topological edge states (TES) excited by left (right)-circularly polarized light, while the topological modes excited by the right(left)-circularly polarized light are forbidden. This is promising for the transmission of the light with Möbius ring robustness. The phase transition from the topological edge state to the bulk state can be conveniently realized by controlling circular polarizations for both Hermitian and non-Hermitian cases, providing a new platform for tuning the topological phase. This work provides a novel tuning degree of freedom of Möbius rings and paves the way for studying topological configuration with non-Euclidean structures. Moreover, the applications of 8PMR are not limited to those investigated in this work. Novel Möbius laser, the tuning of topological invariant by Möbius rings, and more complex non-Hermitian Möbius conditions, can also be explored in the future, which will greatly enrich the non-Euclidean photonic world.

### Supplementary Information

Below is the link to the electronic supplementary material.

Supplementary file1 (PDF 871 KB)

## Data Availability

The data that support the findings of this study are available from the corresponding author, upon reasonable request.
