# Supplementary material for: Spin-controlled topological phase transition in non-Euclidean space
Source: Front Optoelectron. 2024 Mar 19;17(1):7. doi: 10.1007/s12200-024-00110-w (PMC10951149; doi:10.1007/s12200-024-00110-w)
Supplement: Supplementary file 1 — Supplementary file1 (PDF 871 KB) [file 12200_2024_110_MOESM1_ESM.pdf]

Supplementary Materials

Spin-controlled topological phase transition in non-Euclidean space

Zhuochen Du[1] †, Jinze Gao[1]†, Qiuchen Yan[1*], Cuicui Lu[2*], Xiaoyong Hu[1,3,4,5*], Qihuang Gong[1,3,4,5]

[1]State Key Laboratory for Mesoscopic Physics & Department of Physics, Collaborative Innovation Center of Quantum Matter and Frontiers Science Center for Nano-optoelectronics, Beijing Academy of Quantum Information Sciences, Peking University, Beijing 100871, China

[2]Key Laboratory of Advanced Optoelectronic Quantum Architecture and Measurements of Ministry of Education, Beijing Key Laboratory of Nanophotonics and Ultrafine Optoelectronic Systems, School of Physics, Beijing Institute of Technology, Beijing 100081, China

[3]Peking University Yangtze Delta Institute of Optoelectronics, Nantong 226010, China

[4]Collaborative Innovation Center of Extreme Optics, Shanxi University, Taiyuan 030006, China

[5]Hefei National Laboratory, Hefei 230088, China

Corresponding author:

qiuchenyan@pku.edu.cn, cuicuilu@bit.edu.cn, xiaoyonghu@pku.edu.cn

Contents

I. Breaking the degeneracy of TE and TM modes

II. Non-adiabatic-evolution Möbius ring

III. Calculation for Möbius ring as the link ring in SSH model

IV. Calculation for Möbius ring as the link ring in CROW arrays

V. Calculation for regular ring as the link ring in add-drop type micro rings

VI. Band structure for Möbius ring as the link ring in both x and y direction

I. Breaking the degeneracy of TE and TM modes

In the main text, it mentions that even the spin-lock effect works in theory, the simulation results show that such a Möbius ring is still unable to achieve spin lock. We have simulated the 8-π period Möbius ring (8PMR) with the same sizes of ring's length and width. The results are shown in **Figure S1**. The input light is in a $[1, -i]$ form from the upper position of the ring, and four monitors are placed at the symmetrical positions, including the top, bottom, left and right respectively, of the ring. We select two monitors on the bottom (Figure S1(a)) and right positions (Figure S1(b)) of the ring. The bottom monitor demonstrates the anti-clockwise phase distributions along the propagation direction (Ex) and the right monitor demonstrates the clockwise phase distributions along the propagation direction (Ey). Due to the different spin directions of the light, this Möbius cannot play the spin lock function. This is caused by the TE mode and TM mode degeneration as the sizes of length and width in 8PMR are equal. The two modes cannot be distinguished during the ring twist process. To break the degeneracy, we therefore design that the length and width of 8PMR change adiabatically during the twist process.

The results of a straight waveguide with length and width changing adiabatically and twist operation are shown in **Figure S2(a)**. It shows that in this case, the TE mode and

TM mode can be transformed into each other along the propagation direction. We also compare it with a regular waveguide without the length and width adiabatically changing shown in **Figure S2(b)**. The results show that in this case, the modes cannot be transformed along the propagation direction. As a consequence, the adiabatically changing of length and width of 8PMR is necessary and essential.

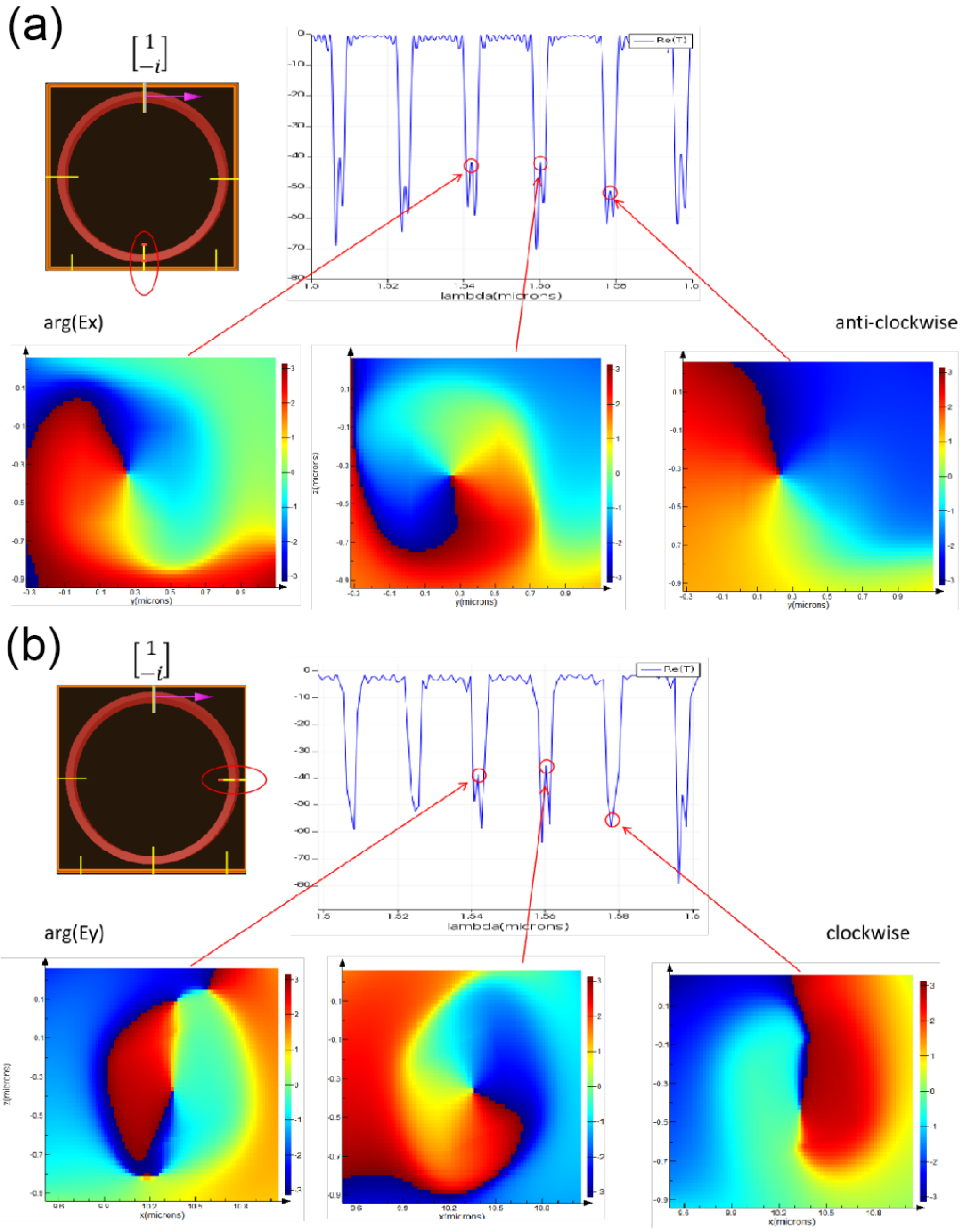

Figure S1 The simulation results of degeneracy between TE and TM modes in a 8-π period Möbius ring without the length and width changing. There is no spin-lock effect. (a) The data obtained from the bottom monitor, showing the anti-clockwise phase distributions. (b) The data obtained from the right monitor, showing the clockwise phase distributions.

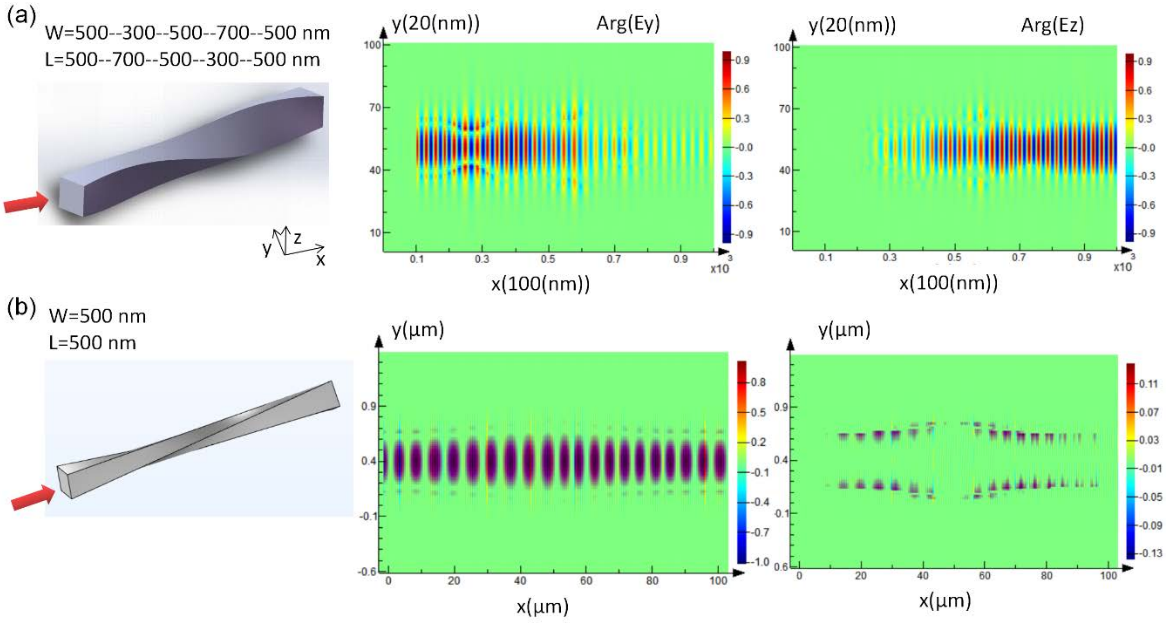

Figure S2 The simulation results of breaking the TE and TM modes degeneracy. (a) The straight waveguide with adiabatically changing of length and width and twist operation. The TE mode (see Arg(Ey)) can convert into TM mode (see Arg(Ex) along the propagation direction. (b) The regular straight waveguide with twist operation. The TE mode (see Arg(Ey)) and TM mode (see Arg(Ex) cannot be transformed into each other along the propagation direction.

II. Non-adiabatic-evolution Möbius ring

If the diameter of a Möbius ring is not large enough, the adiabatic process will be affected. In this case, the light-lock effect still works, but the locking light is not just the circular polarized light, there will be other direction polarized light locking. We have verified the light-lock phenomenon by simulation as shown in **Figure S3**. This Möbius ring has diameter size of 6 um, and the input light is in the $[1, 0]$ form, which represents linear polarized light, from the right position of the ring. Due to the non-adiabatic evolution of the Möbius waveguide ring, the monitor shows that locked light is also linear polarized light instead of the circular polarized light.

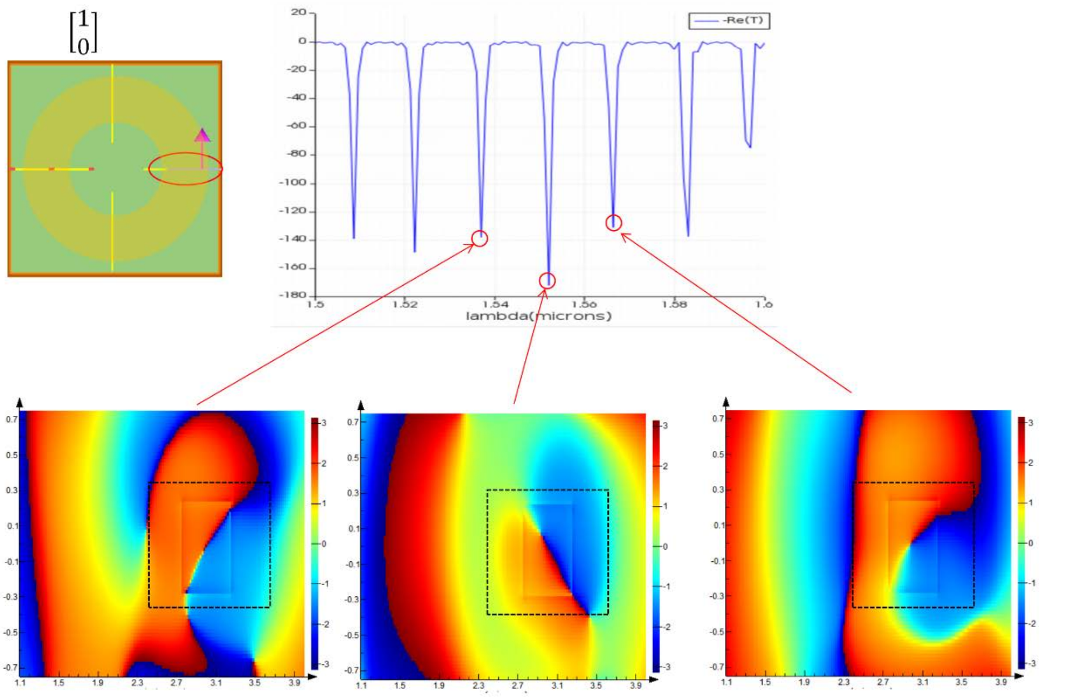

Figure S3 The linear polarized locked light in a non-adiabatic-evolution Möbius ring.

We therefore analyzed the influence of non-adiabatic process on the phases in Möbius ring. We found that an induced extra phase δ factor could well describe this phenomenon. In this case, the Eq.2 can be transformed into Equation S1 as below.

(S1)

$$b = -a * e^{i(\theta_0+\delta)}$$

$$a = b * e^{i\theta_0} = -a * e^{i(2\theta_0+\delta)}$$

Then, the parameter θ_0 needs to meet the following condition, i.e.,

$$\theta_0 = -\frac{1}{2}\delta + \left(n + \frac{1}{2}\right)\pi, n \in Z + \tag{S2}$$

Therefore, we can simplify the original mode of $[a;\ b]$ as $[1;\ i * (-1)^n * e^{-\frac{i}{2}\delta}]$. If the value of δ is zero, the situation is the same as the description in the main text, which represents the adiabatic evolution occurring in a Möbius ring. If the value of δ is π, the supported mode becomes [1, $(-1)^n$], which represents the linear polarized light. In this case, the locked light will then be the linearly polarized light as **Figure S3** shows. If the value of δ is other quantity, it is difficult to judge the specific polarized direction, and the locked light might be some elliptical polarized light. In the main text, we reckon the Möbius rings have undergone adiabatic evolution, therefore, they all have the spin-lock property.

III. Calculation for Möbius ring as the link ring in SSH model

As **Figure 2(a)** shows in the main text, 8PMR is as a link ring in add-drop type micro rings (unit in SSH). Here, we demonstrate more concretely in **Figure S4**. In a unit, the coupling strength is equal as k_1, and the coupling strength is k_2 between units. The dynamic phase factor of the half cycle accumulation in the link ring (Möbius) is also considered to be the same, represented by θ', i.e., $\theta' = \theta - \theta'$. For transmission matrix method (TMM), there can be four matrices to express the process.

$$\begin{bmatrix} d_i \\ b_i \end{bmatrix} = \begin{bmatrix} [\alpha Y e^{i\theta'}]^{-1} & 0 \\ 0 & \alpha Y e^{i\theta'} \end{bmatrix} \begin{bmatrix} a_i \\ c_i \end{bmatrix}$$

(S3)

$$\begin{bmatrix} a_i \\ c_i \end{bmatrix} = \begin{bmatrix} M11 & M12 \\ M21 & M22 \end{bmatrix} \begin{bmatrix} h_i \\ f_i \end{bmatrix}$$

$$\begin{bmatrix} h_i \\ f_i \end{bmatrix} = \begin{bmatrix} [\alpha Y e^{i\theta'}]^{-1} & 0 \\ 0 & \alpha Y e^{i\theta'} \end{bmatrix} \begin{bmatrix} e_i \\ g_i \end{bmatrix}$$

$$\begin{bmatrix} e_i \\ g_i \end{bmatrix} = \begin{bmatrix} M11 & M12 \\ M21 & M22 \end{bmatrix} \begin{bmatrix} d_{i+1} \\ b_{i+1} \end{bmatrix}$$

Where α represents the intrinsic loss of light propagating half cycle in a link ring (Möbius ring), and the value is the same in the main ring (regular ring). The symbol Y represents the Möbius characteristics, i.e., the twist operation, and it can be written as a matrix of $\frac{\sqrt{2}}{2}\begin{bmatrix} 1 & -1 \\ 1 & 1 \end{bmatrix}$. For matrix $\begin{bmatrix} M11 & M12 \\ M21 & M22 \end{bmatrix}$, it is derived from the coupling relationship in add-drop type micro rings. The coupling relationship with regular ring as the link ring in add-drop type micro rings is shown in Section V, and the coupling relationship with Möbius ring as the link ring in add-drop type micro rings has the similar derivation process, but the coupling coefficient k and transmission coefficient t need to be expressed by matrices.

$$k = \begin{bmatrix} k_1 & 0 \\ 0 & k_1 \end{bmatrix} \tag{S4}$$

$$t = \begin{bmatrix} t_1 & 0 \\ 0 & t_1 \end{bmatrix}$$

$$\begin{bmatrix} E_d \\ E_t \end{bmatrix} = \begin{bmatrix} R_x & T_x' \\ T_x & R_x' \end{bmatrix} \begin{bmatrix} E_i \\ E_a \end{bmatrix}$$

$$R_x = -\alpha k_1 Y U^{-1} k_1 e^{i\theta'}$$

$$T_x' = t - \alpha^2 k_1 Y U^{-1} t_1 Y k_1 e^{i\theta}$$

$$T_x = T_x'$$

$$R_x' = R_x$$

$$U = 1 - \alpha^2 t_1 Y t_1 Y e^{i\theta}$$

We can therefore obtain the expression of transmission matrix as

$$\begin{bmatrix} M11 & M12 \\ M21 & M22 \end{bmatrix} = \begin{bmatrix} {R_x}^{-1} & -{R_x}^{-1} * T_x \\ T_x * {R_x}^{-1} & -T_x * {R_x}^{-1} * T_x + {R_x}^{-1} \end{bmatrix} \tag{S5}$$

From the above derivation, we can obtain the total transmission matrix as

$$\begin{bmatrix} d_i \\ b_i \end{bmatrix} = M \begin{bmatrix} d_{i+1} \\ b_{i+1} \end{bmatrix} = e^{-ik_x} \begin{bmatrix} d_{i+1} \\ b_{i+1} \end{bmatrix} \quad \text{(S6)}$$

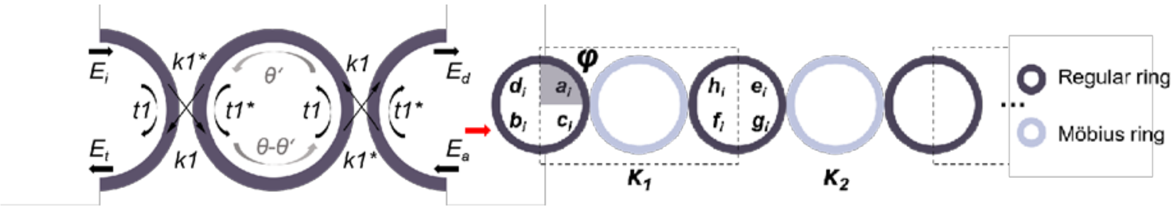

Figure S4 The further schematic of Möbius ring as the link ring in SSH model.

Furthermore, by using the same transmission relationship, the eigenvalue distributions of SSH chains with finite length, as well as the corresponding bulk states and topological edge states can also be solved as shown in **Figure 2(e)-2(h)** in the main text.

IV. Calculation for Möbius ring as the link ring in CROW arrays

The coupling relationship with Möbius ring as the link ring in CROW arrays is similar to the that in SSH model, but there should be both X and Y directions coupling relationship for CROW arrays. As shown in **Equation S4**, the X-direction coupling relationship is the same as the SSH case because the Möbius ring is as the link ring along the X direction. The Y-direction coupling relationship could be simpler because the regular rings are as the link rings along the Y direction (This part can refer to Section V). We therefore can obtain as follows:

$$\begin{bmatrix} d_{i+1} \\ b_{i+1} \end{bmatrix} = M_x M_y \begin{bmatrix} d_i \\ b_i \end{bmatrix} = e^{ik_x} \begin{bmatrix} d_i \\ b_i \end{bmatrix} \quad \text{(S7)}$$

$$M_x = \begin{bmatrix} {R_x}^{-1} & -{R_x}^{-1} * T_x \\ {R_x}^{-1} * T_x & R_x - {R_x}^{-1} * T_x * T_x \end{bmatrix}$$

$$M_y = \begin{bmatrix} {T_y}^{-1} * e^{-2i\varphi} & -{T_y}^{-1} * R_y * e^{-ik_y} \\ {T_y}^{-1} * R_y * e^{ik_y} & (T_y - {T_y}^{-1} * R_y * R_y) * e^{2i\varphi} \end{bmatrix}$$

$$R_y = -\alpha k_1 Y_0 {U_0}^{-1} k_1 e^{i\theta\prime}$$

$$T_y = t - \alpha^2 k_1 Y_0 {U_0}^{-1} t_1 Y k_1 e^{i\theta}$$

$$Y_0 = \begin{bmatrix} 1 & 0 \\ 0 & 1 \end{bmatrix}$$

$$U_0 = 1 - \alpha^2 t_1 Y_0 t_1 Y_0 e^{i\theta}$$

By using the TMM, we can then obtain the corresponding energy band structures. Moreover, by using the similar transmission relationship, the projective band structures and the mode distributions can be obtained as shown in **Figure 3** and **Figure 4** in the main text.

V. Calculation for regular ring as the link ring in add-drop type micro rings

In section III、IV, we have discussed the coupling relationship in SSH model and CROW arrays based on the Möbius ring as the link ring in the X direction. These derivations are on account of the coupling relationship of regular coupled resonant rings. Here, we will briefly show the relationships and more information can refer to reference. The racetrack shaped resonant ring structure is used here, so that the coupling region can be replaced by two coaxial straight waveguide coupling model. The coupling matrix between two resonant rings can be written as:

$$M = \begin{bmatrix} t & \kappa \\ -\kappa^* & t^* \end{bmatrix} = \begin{bmatrix} \cos(KL_{eff}) & -isin(KL_{eff}) \\ -isin(KL_{eff}) & \cos(KL_{eff}) \end{bmatrix} \quad \text{(S8)}$$

where the t and κ represent the transmittance and coupling coefficient in the coaxial waveguide coupler respectively. These two parameters also have the following constraints: $t^2 + k^2 = 1$. When the dielectric constant and wavelength in the ring are selected, the factor K in the matrix is only related to the gap size of the coupling region,

and the factor L_{eff} is the effective coupling length of the coupling region. By applying the coupling matrix to the add-drop type micro rings, the coupling relationship among E_{add}, E_{drop}, E_{input} and $E_{through}$ can be obtained as follows.

$$\begin{bmatrix} E_d \\ E_t \end{bmatrix} = \begin{bmatrix} R & T' \\ T & R' \end{bmatrix} \begin{bmatrix} E_i \\ E_a \end{bmatrix} = \frac{1}{1 - t^2\alpha^2 e^{i\theta}} \begin{bmatrix} -kk^*\alpha e^{i\theta'} & t(1-\alpha^2 e^{i\theta}) \\ t(1-\alpha^2 e^{i\theta}) & -kk^*\alpha e^{i(\theta-\theta')} \end{bmatrix} \begin{bmatrix} E_i \\ E_a \end{bmatrix}$$

Where the intrinsic loss of light propagating half cycle in a link ring is α and the coupling region of the ring is symmetrical. θ represents the phase accumulation in the link ring. θ' represents the phase accumulation in the upper half (divided by the coupling region) of the link ring. Up till now, all the parameters in the transmission matrix become physical quantities that only depend on the optical frequency, and we can further fit the simulation results of an actual configuration with specific structural parameters, all coupling parameter values corresponding to the configuration can be obtained.

VI. Band structure for Möbius ring as the link ring in both x and y direction

In section VI, we have calculated the projective band structure for Möbius ring as the link ring in both x and y direction, and compared it to the case where Möbius ring as the link ring in x direction.

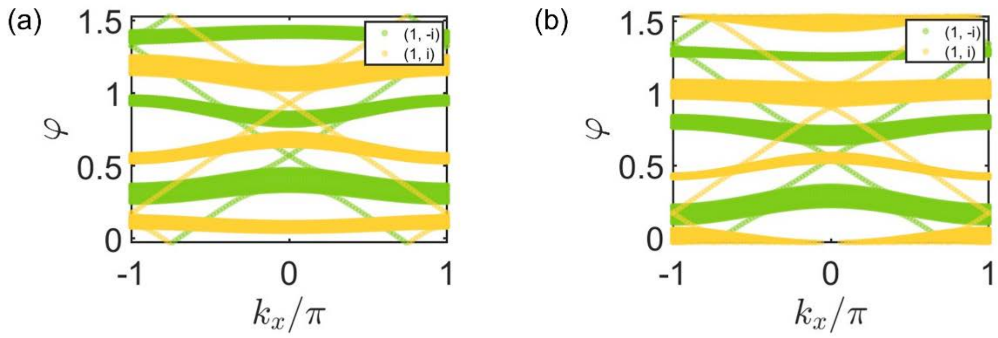

Figure.S5 (a) the projective band structure for Möbius ring as the link ring in x direction. (b) the projective band structure for Möbius ring as the link ring in both x and y direction.

As shown in Figure S5, when Möbius rings are the link ring in both x and y direction, the positions of bands of RCP and LCP will be changed, and there will still be spin-locked effect.

[S1]Y. T. Ao, X. Y. Hu, C. Li, Y. L. You, and Q. H. Gong, Phys Rev Mater **2**, 105201 (2018).
